# Bilateral Medial Tibial Plateau Stress Fractures: A Case Report

**DOI:** 10.7759/cureus.82755

**Published:** 2025-04-21

**Authors:** Mohamed E Eldesouky, Hanan A Bahaaeldin, Baher M Abdelfattah, Adeel Nawaz

**Affiliations:** 1 Department of Trauma and Orthopedics, Dubai Health, Rashid Hospital, Dubai, ARE; 2 Department of Diagnostic Radiology, Zagazig University, Zagazig, EGY

**Keywords:** bilateral, military, proximal tibia, stress fracture, transverse sclerotic metaphyseal bands

## Abstract

Medial tibial plateau stress fractures are rare and often misdiagnosed due to nonspecific symptoms and initially normal radiographs. This case report describes a rare instance of bilateral medial tibial plateau stress fractures in a 43-year-old overweight male police officer with comorbidities including diabetes. The patient developed progressive knee pain after abruptly increasing his running activity. Initial radiographs were inconclusive, but magnetic resonance imaging (MRI) revealed bone marrow edema and non-displaced fractures, highlighting the importance of advanced imaging for early diagnosis. Risk factors included his sudden activity change, high body mass index (BMI), and lower-normal vitamin D levels. Management involved conservative measures like activity modification, weight-bearing as tolerated, and vitamin D supplementation, leading to symptom resolution within three months. The report emphasizes the need for clinical suspicion in middle-aged individuals with persistent knee pain, particularly those with recent activity changes, and underscores MRI's role in early detection. Conservative treatment and patient education on gradual training progression are crucial for recovery and prevention of recurrence.

## Introduction

Stress fractures are a well-recognized form of overuse injury, resulting from repetitive microtrauma that exceeds the bone’s reparative capacity [1-2]. These injuries are generally classified into two categories: fatigue fractures and insufficiency fractures. Fatigue fractures occur in otherwise healthy bone subjected to abnormal or repetitive loading. They are commonly seen in younger individuals engaged in high-impact activities such as running, jumping, or marching. In contrast, insufficiency fractures occur when normal loading is applied to weakened bone, seen in elderly patients with osteoporosis or younger patients with chronic corticosteroid use [3].

Fatigue fractures are commonly observed in athletes and military personnel, particularly in the tibia, fibula, metatarsals, and calcaneus. Epidemiological studies suggest that stress fractures account for up to 20% of sports injuries [4]. While the tibial shaft is the most common site for stress fractures, involvement of the medial tibial plateau is relatively rare [5].

Diagnosis can be challenging, as symptoms often mimic other conditions such as meniscal injuries, periostitis, pes anserinus bursitis, and osteochondral lesions. Initial radiographs are usually inconclusive in the early stages, making advanced imaging modalities such as MRI and bone scintigraphy crucial for early detection. MRI, in particular, is highly sensitive and specific for detecting bone marrow edema and periosteal reactions before cortical disruption becomes evident on radiographs [5-6].

In this case report, we present a rare case of bilateral medial tibial plateau stress fractures, a condition that has been sparsely reported in the literature. We aim to discuss the diagnostic challenges, imaging findings, and management strategies to enhance awareness of this uncommon but significant injury.

## Case presentation

A 43-year-old male of Arab origin, a police officer with a medical history of diabetes mellitus, hyperlipidemia, chronic prostatitis, and non-alcoholic steatohepatitis, was referred to our clinic for progressive bilateral knee pain. His anthropometric measurements were notable for a height of 169 cm and a weight of 86 kg, placing him in the overweight BMI range. His medications included pioglitazone, alfuzosin, rosuvastatin, semaglutide, mirabegron, and vitamin D supplementation.

**Figure 1:**
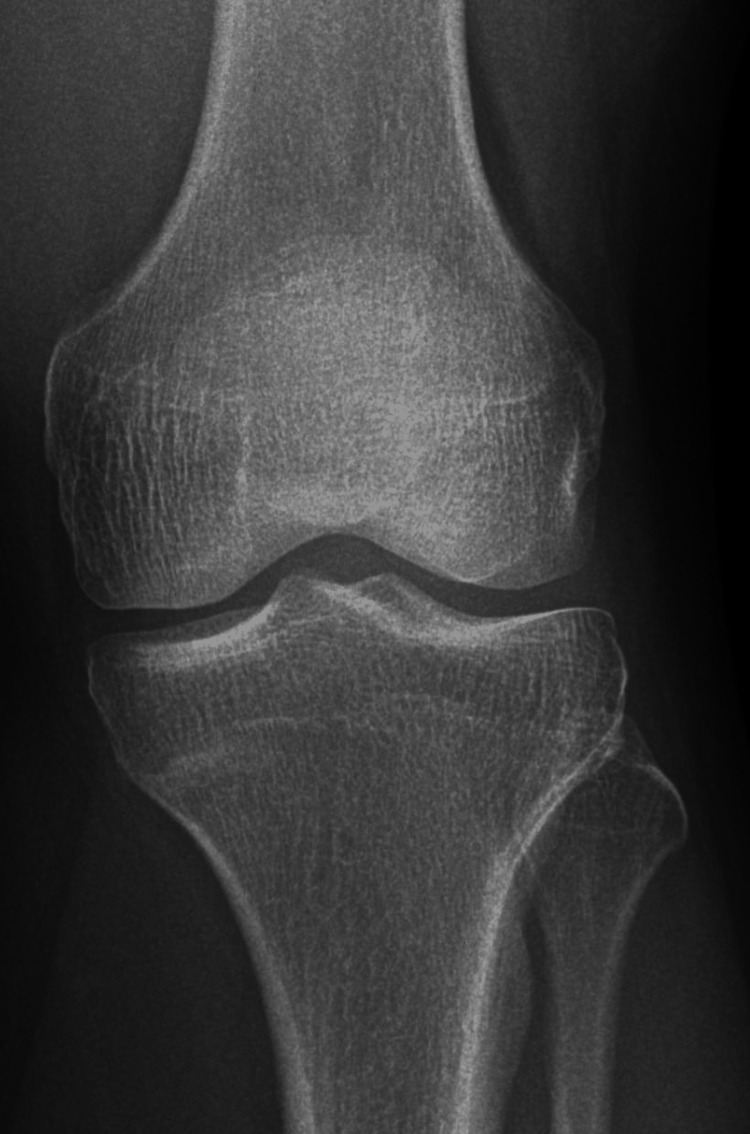
X-ray AP view of left knee showing unremarkable findings

**Figure 2:**
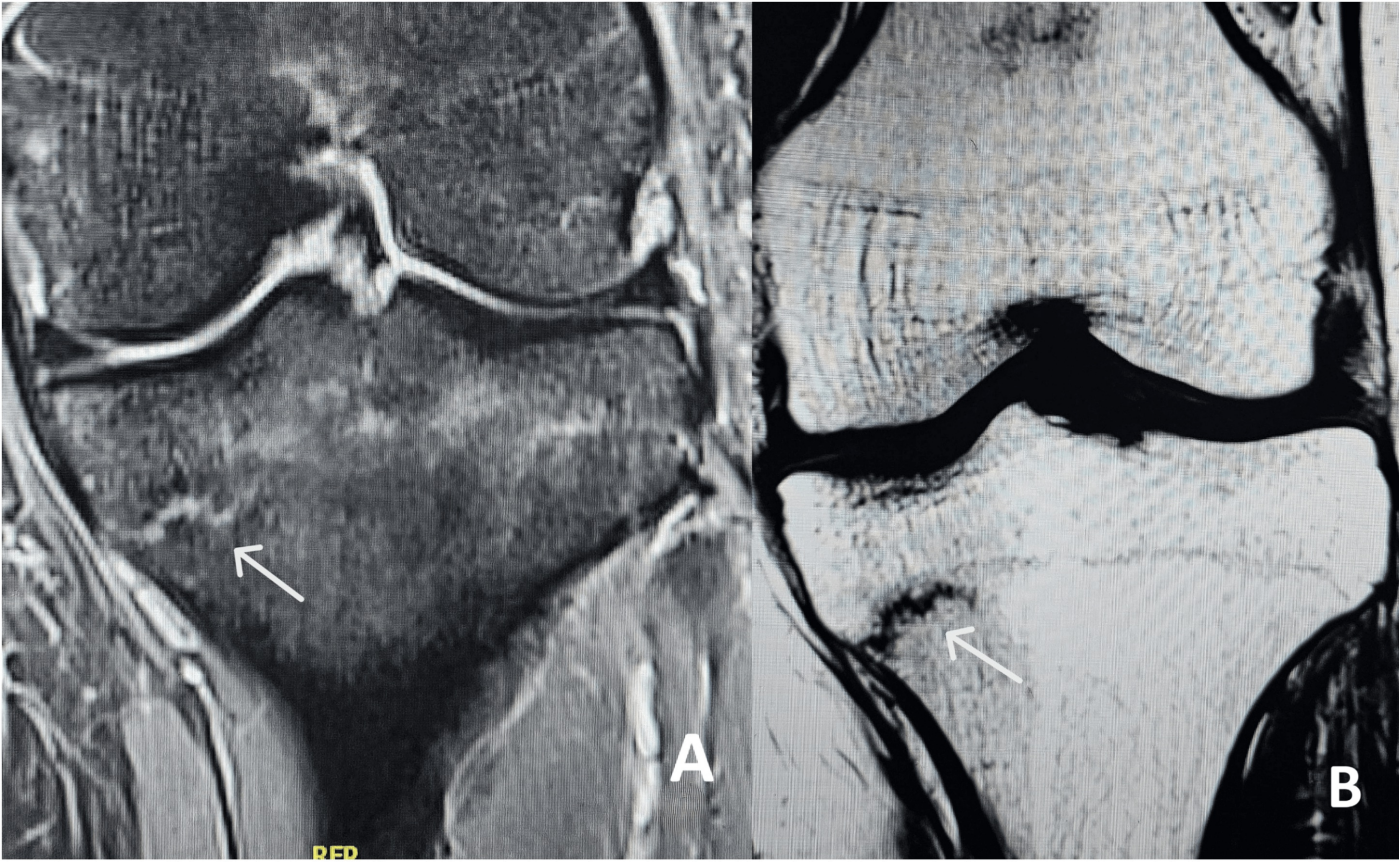
MRI of left knee short tau inversion recovery (STIR) (A) and T1-weighted images (B) showing medial proximal tibia stress fracture (white arrow)

The patient's symptoms began six months prior, with intermittent left knee pain that developed insidiously at home, without any preceding trauma. Initial radiographs were unremarkable (Figure 1). At the time, his family physician suspected a meniscal tear or ligament sprain and ordered an MRI. Laboratory tests revealed a vitamin D level of 31.9 ng/mL, within the lower normal range. MRI showed a non-displaced transverse fracture of the proximal medial tibia with associated bone marrow edema, raising suspicion for a stress fracture (Figure 2). Following this, he was referred to our clinic.

The patient noted progression of pain, which became bilateral. His history revealed a recent increase in physical activity, as he had begun running approximately 3 km daily on paved roads for two to three weeks before symptom onset, as part of his training. Despite his symptoms, the physical examination findings were largely unremarkable. He had a full range of motion with no visible deformity, ecchymosis, or significant swelling. There was mild tenderness over the proximal tibia bilaterally, but no joint line tenderness. Physical examination revealed a full range of motion without effusion or instability. Negative patellar grind, drawer, and valgus/varus stress tests ruled out ligamentous or patellofemoral pathology. Tenderness localized to the proximal medial tibia, absent joint line tenderness, further distinguished this from meniscal injury.

**Figure 3:**
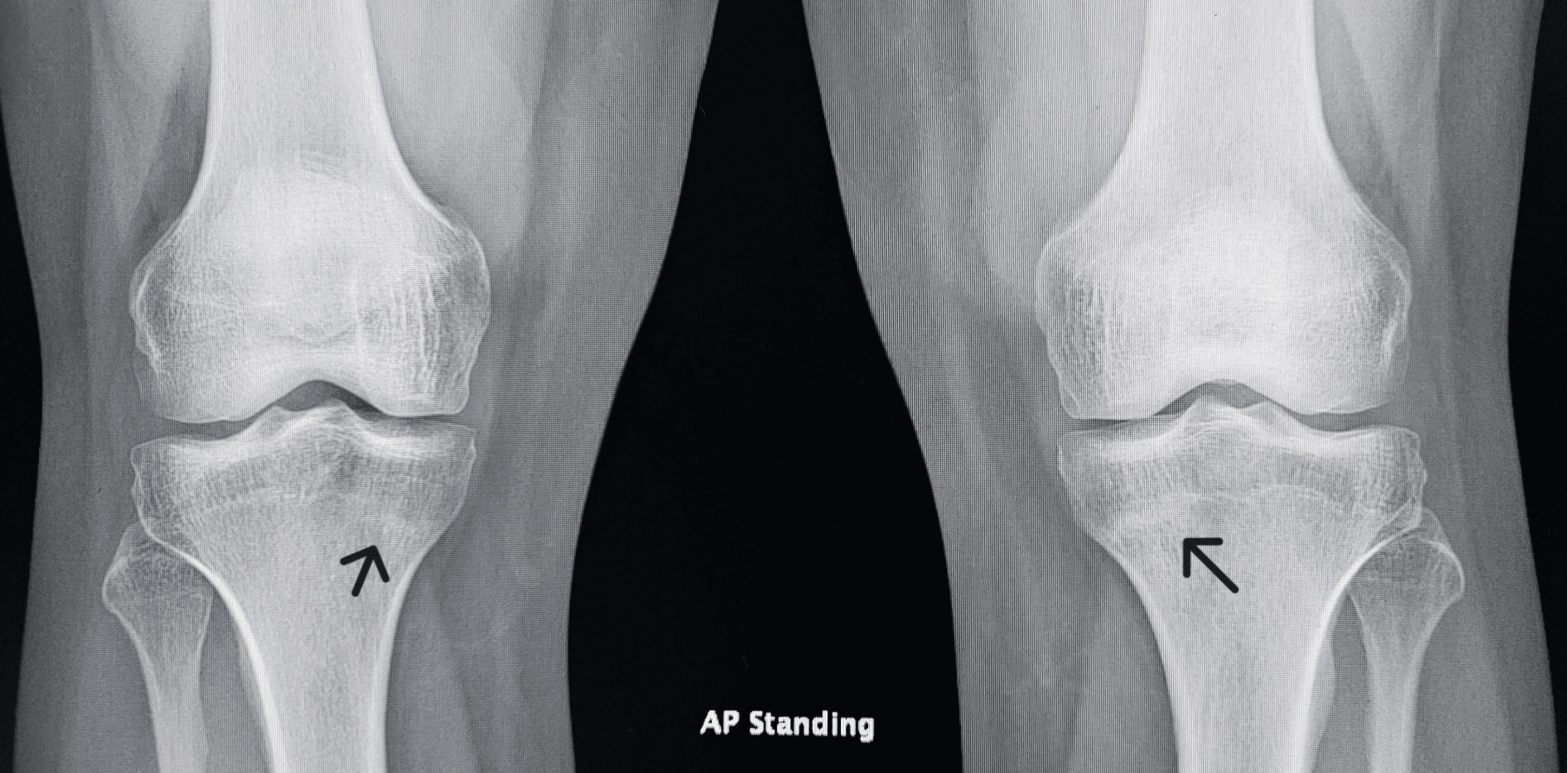
Three-month follow-up X-ray AP view of both knees showing bilateral sclerotic bands at the medial proximal tibiae (black arrow)

Given the clinical presentation, follow-up radiographs after three months revealed subtle linear sclerosis at the proximal tibia bilaterally (more pronounced on the left) (Figure 3), further supporting the diagnosis of stress fractures. MRI of the contralateral knee was recommended for further evaluation. The patient was also advised to continue full weight-bearing mobilization and to avoid any vigorous or high-impact activities or exercises, in addition to calcium and vitamin D supplements.

**Figure 4:**
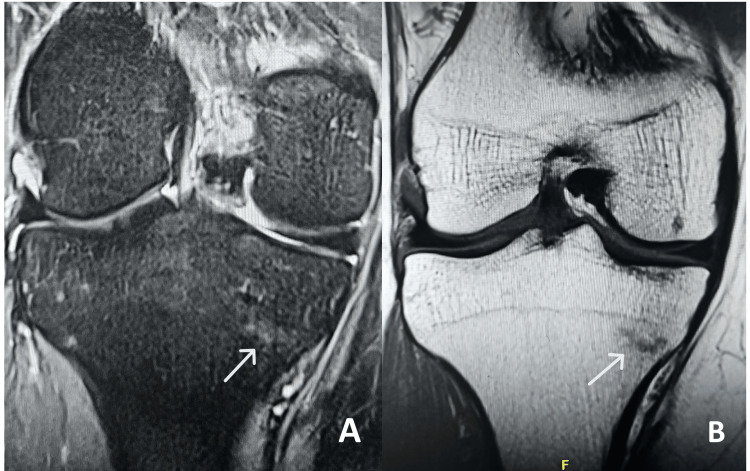
MRI of right knee short tau inversion recovery (STIR) (A) and T1-weighted images (B) showing medial proximal tibia stress fracture (white arrow)

Subsequent MRI of the right knee confirmed bilateral stress fractures of the proximal tibiae (Figure 4). On follow-up, three months later, the patient reported significant symptom improvement with conservative management and demonstrated no residual tenderness or functional limitations.

## Discussion

Stress fractures are overuse injuries that occur when repetitive mechanical loading exceeds the bone’s natural remodeling capacity. They are classified into fatigue fractures, which develop in normal bone subjected to abnormal stress, and insufficiency fractures, which occur in weakened bone under normal stress [3]. Our patient, a 43-year-old male with multiple comorbidities, including diabetes mellitus, chronic prostatitis, and non-alcoholic steatohepatitis, developed bilateral proximal tibial stress fractures - a pattern more commonly associated with high-impact athletic activity or intense repetitive loading.

The pathophysiology of stress fractures involves an imbalance between osteoclastic resorption and osteoblastic repair in response to repetitive mechanical stress. Initially, increased osteoclastic activity leads to trabecular microfractures. This process is accompanied by the replacement of circumferential lamellar bone with dense osteonal bone, along with bone marrow edema detectable on MRI. If repetitive stress continues without adequate rest, these microfractures can coalesce, ultimately leading to a complete cortical break [1-2].

Several risk factors influence the development of stress fractures, including training errors, biomechanical abnormalities, metabolic deficiencies, and hormonal imbalances [7]. Runners, for instance, experience stress fractures in 13-37% of cases, with an increased incidence when weekly running distances exceed 32 km [8-9]. Research by Milgrom et al. demonstrated that axial compressive and tensile forces increase by up to 285% during overground running compared to treadmill running [10]. Additionally, McKenzie et al. found that foot-strike mechanics, particularly slight pronation during ceremonial marching, amplify stress on the medial knee compartment [11]. While this case report identifies abrupt activity increase and metabolic factors as key contributors, biomechanical assessments (e.g., gait analysis, foot pronation) were not performed. Future studies should evaluate running mechanics in similar cases to delineate their role in medial tibial plateau stress fractures.

The evolution of his symptoms, progressing to bilateral involvement and eventual radiographic changes, highlights the dynamic nature of stress fractures when repetitive loading continues unchecked [7]. Multiple factors likely predisposed this patient to injury. His sudden increase in physical activity, combined with a high BMI, resulted in excessive axial loading on the tibiae. His vitamin D level of 31.9 ng/mL, though within the lower normal range, may have further impaired bone remodeling. Additionally, chronic disease and medication effects, particularly the use of pioglitazone for diabetes, have been associated with osteopenic changes, further increasing fracture susceptibility [12]. Studies emphasize that most stress fractures develop four to five weeks after a significant change in training intensity. Furthermore, muscular fatigue compromises the ability of muscles to absorb mechanical stress, transmitting greater forces to the underlying bone and facilitating injury [6,13]. This case underscores the importance of a detailed history, particularly regarding activity changes and underlying health conditions, as these factors are among the most critical in identifying bone stress injuries.

Tibial stress fractures are frequently observed in military recruits and runners but typically involve the tibial shaft rather than the proximal tibial plateau [5]. When they do occur in the proximal tibia, they tend to localize to the posteromedial aspect, as this is the region of maximal stress, as described in the literature and observed in this case [14]. The location of stress fractures in runners varies with age, while younger athletes are more prone to fibular and tibial shaft fractures, older individuals are more likely to sustain femoral and tarsal stress fractures [15]. However, bilateral medial tibial plateau stress fractures are exceedingly rare. Prior reports by Engber and Harolds documented similar cases in military personnel, often misdiagnosed as meniscal injuries, pes anserinus bursitis, or ligamentous sprains - conditions that were initially considered in our patient’s differential diagnosis [14,16].

The patient’s tenderness along the medial joint line, combined with his clinical history, initially raised suspicion for a meniscal or soft tissue injury. This diagnostic pitfall is common, as early stress fractures are often radiographically occult, particularly within the first two to three weeks of symptom onset. MRI plays a pivotal role in early diagnosis and is considered more sensitive than CT and more specific than scintigraphy, as focal tracer uptake can occur with any process that stimulates bone remodeling, including tumors, infections, or stress reactions without fracture [5-6,17]. T2-weighted and short tau inversion recovery (STIR) sequences are particularly useful for detecting bone marrow edema, a hallmark of early stress injury. Unlike stress fractures in cortical bone, which often present with periosteal reactions, those in cancellous bone, such as the tibial metaphysis, typically exhibit a sclerotic band due to trabecular collapse and repair, a key finding in this patient’s imaging [5-6].

Despite the potential severity of stress fractures, they generally heal well with conservative management. Treatment focuses on rest, load modification, and correction of metabolic deficiencies to support bone healing [7]. Medial tibial plateau stress fractures are primarily compression-type injuries. Chiba et al. describe anterior tibial plateau fractures as resulting from forces exerted by the medial femoral condyle during hyperextension and varus loading [18]. The distinction between compression- and tension-type stress fractures is clinically significant, as compression-type fractures heal well with conservative treatment due to the creation of an electronegative environment that stimulates osteoblast activity. In contrast, tension-type fractures, such as those occurring in the lateral superior cortex of the femoral neck, create an electropositive environment that stimulates osteoclastic resorption, leading to an increased risk of complications and often requiring surgical fixation [19-20].

Early recognition through appropriate imaging and clinical follow-up prevented complications in this case, with symptom resolution confirmed at follow-up. Treatment was conservative, focusing on temporary activity modification rather than strict immobilization, as previous studies have shown that complete rest may lead to poor compliance in physically active individuals. Engber reported that maintaining light activity enhances patient adherence and promotes recovery, as pain naturally limits activity without the need for prolonged immobilization [16]. The patient was advised to gradually resume weight-bearing activities as tolerated, ensuring a progressive return to function while minimizing the risk of recurrence.

## Conclusions

This case highlights the need for heightened clinical suspicion when evaluating persistent knee pain in middle-aged individuals, particularly those with recent changes in activity levels. A thorough history, focusing on activity patterns, metabolic health, and biomechanical risk factors, is essential for accurate diagnosis and optimal management. MRI remains the gold standard for early detection, especially when radiographs appear normal. Lastly, stress fractures should be considered in middle-aged, overweight patients with persistent knee pain, particularly after sudden activity changes. MRI is indispensable for early diagnosis and conservative management, including load modification and pain-free weight bearing, and can ensure full recovery.
